# Gestational age and hospital admission costs from birth to childhood: a population-based record linkage study in England

**DOI:** 10.1136/archdischild-2022-324763

**Published:** 2023-02-09

**Authors:** Xinyang Hua, Stavros Petrou, Victoria Coathup, Claire Carson, Jennifer J Kurinczuk, Maria A Quigley, Elaine Boyle, Samantha Johnson, Alison Macfarlane, Oliver Rivero-Arias

**Affiliations:** 1 Centre for Health Policy, Melbourne School for Population and Global Health, University of Melbourne, Melbourne, Victoria, Australia; 2 National Perinatal Epidemiology Unit, Nuffield Department of Population Health, University of Oxford, Oxford, UK; 3 Nuffield Department of Primary Care Health, University of Oxford, Oxford, UK; 4 Department of Health Sciences, University of Leicester, Leicester, UK; 5 Centre for Maternal and Child Health Research, City, University of London, London, UK

**Keywords:** Health services research, Neonatology, Child Health, Health Care Economics and Organizations

## Abstract

**Objective:**

To examine the association between gestational age at birth and hospital admission costs from birth to 8 years of age.

**Design:**

Population-based, record linkage, cohort study in England.

**Setting:**

National Health Service (NHS) hospitals in England, UK.

**Participants:**

1 018 136 live, singleton births in NHS hospitals in England between 1 January 2005 and 31 December 2006.

**Main outcome measures:**

Hospital admission costs from birth to age 8 years, estimated by gestational age at birth (<28, 28–29, 30–31, 32, 33, 34, 35, 36, 37, 38, 39, 40, 41 and 42 weeks).

**Results:**

Both birth admission and subsequent admission hospital costs decreased with increasing gestational age at birth. Differences in hospital admission costs between gestational age groups diminished with increasing age, particularly after the first 2 years following birth. Children born extremely preterm (<28 weeks) and very preterm (28–31 weeks) still had higher average hospital admission costs (£699 (95% CI £419 to £919) for <28 weeks; £434 (95% CI £305 to £563) for 28–31 weeks) during the eighth year of life compared with children born at 40 weeks (£109, 95% CI £104 to £114). Children born extremely preterm had the highest 8-year cumulative hospital admission costs per child (£80 559 (95% CI £79 238 to £82 019)), a large proportion of which was incurred during the first year after birth (£71 997 (95% CI £70 866 to £73 097)).

**Conclusions:**

The association between gestational age at birth and hospital admission costs persists into mid-childhood. The study results provide a useful costing resource for future economic evaluations focusing on preventive and treatment strategies for babies born preterm.

**WHAT IS ALREADY KNOWN ON THIS TOPIC**

- Most previous research on the economic consequences associated with gestational age focused on the costs of initial birth admission or costs incurred during the first few years after birth.
- A small number of studies have examined the association between gestational age at birth and hospital costs over the longer term, but were based on regional data, decision-analytic models synthesising summary evidence from multiple sources, cross-sectional assessments at specific ages, or focused on narrow categories of the gestational age range.

**WHAT THIS STUDY ADDS**

- Using a large national cohort with hospital records linked from birth until mid-childhood, our study quantifies hospital admission costs from birth up to 8 years of age across the full range of gestational age at birth.

**HOW THIS STUDY MIGHT AFFECT RESEARCH, PRACTICE OR POLICY**

- The results should act as a useful resource for clinical and budgetary service planning, and as data inputs for economic evaluations of preventive and treatment strategies for babies born at different gestational ages.

## Background

The rates of preterm birth (<37 weeks’ gestation) have increased or remained stable over the past few decades in most countries,^1–3^ accounting for 10.6% of all live births worldwide in 2014.^3^ Survival rates following preterm birth have increased as a result of technological advances,^4 5^ but these babies still remain at a higher risk of infant mortality and a range of short-term and long-term morbidities.^6 7^ A recent study examined the association between gestational age at birth and hospital admissions and found that gestational age at birth is a strong predictor of severe morbidity throughout childhood, even for those born at 38 and 39 weeks’ gestation.^8^

Most previous research on the economic consequences associated with gestational age focused on the costs of initial birth admission or costs incurred during the first few years after birth.^9^ A small number of studies have investigated hospital costs by gestational age over the longer term, but were based on historical region-specific data,^10^ decision-analytic models synthesising summary evidence from multiple sources,^11 12^ cross-sectional assessments at a specific age,^13^ or focused on narrow categories of the gestational age spectrum.^14^ To the best of our knowledge, no study has comprehensively estimated the long-term economic burden associated with gestational age from birth to middle childhood across the full spectrum of gestational age using a large national cohort of children born in the 21st century.

In this study, we conducted an evidence synthesis exercise to examine the association between gestational age at birth and hospital admission costs from birth to 8 years of age using a population-based, record-linkage study that included all live, singleton births occurring in England in 2005/2006 as part of the TIGAR study (Tracking the Impact of Gestational Age on Health, Educational and Economic outcomes: a Longitudinal Records Linkage Study).^8^

## Methods

### Data sources

In this study, we synthesised data from three data sources: TIGAR, National Neonatal Research Database (NNRD) and Paediatric Intensive Care Audit Network (PICANet) databases. Access to individual patient-level data was available for the TIGAR dataset, whereas aggregate data were available from the NNRD and PICANet datasets.^15 16^

The TIGAR cohort was built through a population-based data linkage using data from the Office for National Statistics (ONS) birth registration records linked to death registration records, birth notification records and Hospital Episode Statistics Admitted Patient Care (HES APC) records.^17^ A description of the datasets, linkage and quality assurance has been published elsewhere.^8 18 19^ In brief, the TIGAR cohort included all live, singleton births occurring in an NHS hospital in England between 1 January 2005 and 31 December 2006 with follow-up capturing all inpatient admissions to National Health Service (NHS) hospitals in England from birth until 31 March 2015. Children were not eligible if they had opted out, died before discharge from the birth admission or if there were data quality issues.^8^

The NNRD is a national resource holding quality-assured real-world clinical data captured during the course of care for all admissions to NHS neonatal units in England, Wales, Scotland and the Isle of Man.^15^ PICANet is an audit database recording demographic and clinical information on all patients admitted to paediatric intensive care units in the UK and Ireland.^16^

### Study design

The main source of information for our economic analysis was the TIGAR dataset. However, although in the HES APC records the length of stay for admissions includes any time spent in critical care units, the information did not reliably indicate the level of care the child received on a day-to-day basis (online supplemental material 1.1). Therefore, we requested bespoke aggregate tables from the NNRD and PICANet to supplement the individual-level data within the TIGAR cohort. This information was used together with the total number of live births in England by gestational age in the same year, as reported by ONS, to simulate the following information for children in the TIGAR cohort by sex and gestational age at birth at an individual level^20^:

10.1136/fetalneonatal-2022-324763.supp1 Supplementary data

1. Whether a child was admitted to a neonatal or paediatric critical care unit during the birth admission.
2. The number of days that were spent in a neonatal or paediatric critical care unit during the birth admission by level of care.

Any difference between the total birth admission days observed in the TIGAR cohort and the critical care days estimated from the NNRD and PICANet was considered non-critical care ward days. More details about the methods used to simulate neonatal and paediatric critical care days and calculate non-critical care ward days in birth admission can be found in online supplemental materials 1.2 and 1.3.

### Costs

We estimated direct costs from a healthcare perspective in England. In the HES APC, each data record indicates a Finished Consultant Episode, which represents a continuous period of care under one clinical consultant. Costs were estimated at episode level. The 2018–2019 Casemix Grouper Software (HRG4+) was used to allocate each episode to a Healthcare Resource Group (HRG), primarily based on any procedures performed, diagnoses, hospital admission type, episode length of stay and patient characteristics.^21^ HRGs are standard groupings of clinically similar treatments, which use comparable levels of healthcare resource. The NHS 2017–2018 reference cost schedules were used to price the HRGs.^22^ More details on the application of the Grouper Software and matching the HRGs to reference costs can be found in online supplemental materials 2 and 3.

Neonatal and paediatric critical care costs during the birth admission were calculated on a per diem basis using the NHS 2017–2018 reference costs based on level of care. Birth admission ward costs were adjusted accordingly by extracting days spent in critical care units from the total hospital stay (online supplemental material 1.3).

### Statistical analysis

We used descriptive statistics to estimate the average total length of stay and average costs for the birth admission and for subsequent hospital admissions by year of follow-up among children alive at the beginning and not censored by the end of each follow-up year. Comparisons were made between the following groups by gestational age at birth (weeks): <28, 28–29, 30–31, 32, 33, 34, 35, 36, 37, 38, 39, 40, 41, 42. Gestational ages were grouped <28, 28–29 and 30–31 following ONS policy about reporting of small numbers.

We used the Kaplan-Meier sample average estimator to calculate the total 1-year, 5-year and 8-year costs for each gestational age group.^23^ The 95% CIs for total costs and p values for cost differences between gestational age at birth using 40 weeks as the reference were obtained using non-parametric bootstrapping with 1000 replications. No adjustment based on baseline characteristics was conducted.

In the baseline analysis, we calculated the birth admission non-critical care ward days and costs according to aggregated data from the NNRD and PICANet. We conducted a sensitivity analysis to investigate the results when allocating all the estimated non-critical care ward days to neonatal critical care for children born at ≤33 weeks to reflect an scenario where these babies spent most of their stay in a neonatal unit as previously indicated.^24^

All analyses were conducted using Stata V.14 (College Station, Texas, USA). Costs are presented in 2018 UK pounds (£).

## Results

A total of 1 018 136 children were included in this study, with a total of 9 372 105 person years of follow-up and 1 315 338 admissions that occurred between 1 January 2005 and 31 March 2015. The baseline characteristics of the TIGAR cohort are presented in table 1. Among the 1 018 136 children included in this study, 56 053 (5.5%) were born at <37 weeks, and 99 717 (9.8%) were small for gestational age at birth (birth weight below the 10th centile).

**Table 1:** Baseline characteristics of live born singletons (total N=.1 018 136)

	n	%
Mother’s age at childbirth (years)		
<20	44 486	4.4
20–24	181 633	17.8
25–29	253 055	24.9
30–34	293 741	28.9
35–39	193 622	19.0
40+	51 599	5.1
Parity		
Nulliparous	480 616	47.2
Parous	496 203	48.7
Missing	41 317	4.1
Mother’s marital status		
Married	581 160	57.1
Partner	347 366	34.1
Single	89 610	8.8
Mother’s country of birth		
Non-UK	225 695	22.2
UK	791 012	77.7
Missing	1429	0.1
Index of Multiple Deprivation*		
Least deprived Q1	276 838	27.2
Q2	216 006	21.2
Q3	180 300	17.7
Q4	161 793	15.9
Most deprived Q5	157 195	15.4
Missing	26 004	2.6
Sex		
Male	521 169	51.2
Female	496 967	48.8
Ethnicity (child)		
White British	677 236	66.5
White other	59 683	5.9
Bangladeshi	14 546	1.4
Indian	27 783	2.7
Pakistani	41 739	4.1
Black African	34 571	3.4
Black Caribbean	12 410	1.2
Other	91 570	9.0
Missing	58 598	5.8
Gestational age (weeks)		
<28	1730	0.2
28–29	2089	0.2
30–31	3227	0.3
32	2656	0.3
33	4050	0.4
34	7292	0.7
35	11 663	1.1
36	23 346	2.3
37	54 001	5.3
38	137 926	13.5
39	231 376	22.7
40	288 065	28.3
41	208 757	20.5
42	41 958	4.1
Small for gestational age at birth		
No	918 419	90.2
Yes	99 717	9.8
Mode of birth		
Vaginal	751 653	73.8
Caesarean section	222 615	21.9
Missing	43 868	4.3
Labour induction		
No	626 178	61.5
Yes	154 851	15.2
Missing	237 107	23.3
Season of birth		
Jan–Mar	236 944	23.3
Apr–Jun	254 016	24.9
Jul–Sep	270 282	26.5
Oct–Dec	256 894	25.2

*The Index of Multiple Deprivation (IMD) is the official measure of relative deprivation for small areas (or neighbourhoods) in England.

The proportions of children admitted to neonatal or paediatric critical care during the birth admission are presented in table 2. The admission rates decreased with increasing gestational age at birth, with the lowest rate observed at 39–40 weeks (table 2). Children born extremely preterm (<28 weeks) spent, on average, 50.2 (SD 18.8) days in neonatal critical care, which generated an average cost of £53 144 (SD 15 504); in comparison, 8% of children born at 40 weeks were admitted to neonatal critical care with an average stay of 4.0 (SD 2.5) days, which generated an average cost of £2369 (SD £1783). Further, 5.5% of children born extremely preterm and 0.1% of children born at 40 weeks were admitted to paediatric critical care during their birth admission, which generated an average cost of £14 967 (SD £9526) and £21 957 (SD £14 523), respectively. The average hospital days and costs incurred as a result of admission to non-critical care wards also decreased with increasing gestational age at birth (table 3), with the longest stay and highest cost observed for the extremely preterm group (48.8 days, £13 142) and the shortest stay and lowest cost observed for those born at 40 gestational weeks (1.3 days, £244). The total costs associated with birth admissions including critical and non-critical care is presented in table 4.

**Table 2:** Proportion of children admitted to neonatal critical care (NCC) and paediatric critical care (PCC) during the birth admission and, among those admitted, length of stay and cost, by gestational age at birth

	% to NCC	Days in NCC, mean (SD)	Costs in NCC, mean (SD)	% to PCC	Days in PCC, mean (SD)	Costs in PCC, mean (SD)
<28	100%	50.2 (18.8)	£53 144 (15 504)	5.5%	8.8 (5.9)	£14 967 (9526)
28–29	100%	39.3 (13.8)	£32 717 (10 185)	1.7%	9.6 (4.0)	£18 265 (9166)
30–31	100%	30.2 (9.3)	£21 957 (6753)	1.7%	9.0 (4.0)	£17 596 (8361)
32	100%	22.0 (6.2)	£15 350 (4772)	0.4%	16.1 (6.5)	£28 530 (10 386)
33	100%	16.3 (4.4)	£11 065 (3516)	0.4%	12.5 (7.8)	£24 168 (15 387)
34	90%	11.9 (4.4)	£7883 (3277)	0.4%	13.3 (7.0)	£24 390 (13 400)
35	63%	7.7 (3.9)	£5018 (2981)	0.4%	13.5 (6.6)	£24 087 (13 943)
36	40%	5.7 (3.4)	£3757 (2658)	0.4%	11.2 (6.8)	£20 069 (13 466)
37	20%	5.0 (2.9)	£3343 (2419)	0.2%	9.4 (5.0)	£19 003 (11 033)
38	11%	4.3 (2.8)	£2821 (2302)	0.2%	9.5 (4.9)	£19 225 (11 420)
39	8%	4.0 (2.5)	£2417 (1798)	0.1%	10.6 (5.9)	£23 720 (15 050)
40	8%	4.0 (2.5)	£2369 (1783)	0.1%	9.8 (5.9)	£21 957 (14 523)
41	10%	4.0 (2.4)	£2411 (1721)	0.1%	5.3 (3.7)	£13 080 (12 786)
42	12%	4.1 (2.6)	£2383 (1825)	0.1%	5.5 (3.2)	£10 936 (9374)

**Table 3:** Non-critical ward days and costs during the birth admission, by gestational age at birth

	n	Mean days in non-critical care ward	Mean costs in non-critical care ward	95% CI
<28	1730	48.8	£13 142	£12 512 to £13 771
28–29	2089	23.4	£5567	£5310 to £5823
30–31	3227	9.7	£2394	£2291 to £2498
32	2656	5.4	£1377	£1311 to £1443
33	4050	3.7	£1016	£963 to £1068
34	7292	1.8	£532	£509 to £556
35	11 663	2.4	£809	£775 to £843
36	23 346	2.2	£753	£725 to £781
37	54 001	1.8	£463	£446 to £480
38	137 926	1.8	£373	£365 to £382
39	231 376	1.6	£285	£279 to £290
40	288 065	1.3	£244	£240 to £249
41	208 757	1.4	£262	£258 to £267
42	41 958	1.5	£342	£329 to £356

**Table 4:** Birth admission costs and costs in subsequent admissions by year of follow-up and gestational age

		Birth admission	Year 1	Year 2	Year 3	Year 4	Year 5	Year 6	Year 7	Year 8
<28	n	1730	1730	1710	1705	1701	1697	1695	1695	1691
	Mean	£67 168	£4885	£2726	£1539	£1347	£933	£848	£656	£669
	SD	£33 071	£10 937	£11 342	£6446	£8032	£4895	£7961	£4333	£5248
28–29	n	2089	2089	2071	2069	2067	2067	2066	2065	2064
	Mean	£38 616	£2893	£1410	£716	£703	£567	£587	£564	£522
	SD	£18 893	£7610	£6564	£3033	£4419	£4252	£4289	£5511	£4719
30–31	n	3227	3227	3218	3217	3214	3213	3212	3211	3211
	Mean	£24 640	£2029	£754	£636	£421	£527	£434	£330	£377
	SD	£12 231	£5698	£3309	£5178	£2225	£4082	£4119	£2142	£4805
32	n	2656	2656	2646	2645	2644	2642	2642	2641	2639
	Mean	£16 803	£1813	£842	£592	£539	£442	£370	£344	£240
	SD	£8056	£6004	£4578	£3955	£4104	£2837	£2899	£2094	£1267
33	n	4050	4050	4026	4021	4018	4017	4016	4015	4015
	Mean	£12 212	£1387	£667	£399	£311	£325	£256	£232	£194
	SD	£7316	£5931	£4234	£2819	£1695	£2733	£1571	£1458	£1218
34	n	7292	7292	7255	7247	7244	7242	7240	7240	7240
	Mean	£7732	£1399	£655	£396	£338	£251	£278	£262	£209
	SD	£6570	£7269	£5340	£3547	£3224	£1829	£2982	£2316	£1755
35	n	11 663	11 663	11 614	11 601	11 593	11 590	11 588	11 588	11 583
	Mean	£4052	£1086	£503	£311	£275	£259	£243	£226	£218
	SD	£6990	£5883	£4195	£2625	£2636	£2759	£2801	£2622	£2701
36	n	23 346	23 346	23 273	23 255	23 249	23 246	23 239	23 237	23 235
	Mean	£2325	£1030	£437	£314	£264	£279	£242	£205	£181
	SD	£6235	£6061	£3141	£3468	£2378	£3700	£2913	£2901	£2454
37	n	54 001	54 001	53 889	53 865	53 845	53 836	53 824	53 818	53 812
	Mean	£1166	£744	£356	£247	£233	£201	£196	£167	£159
	SD	£4709	£4012	£2953	£2296	£2516	£1921	£2482	£1677	£1887
38	n	137 926	137 926	137 711	137 654	137 616	137 596	137 575	137 563	137 553
	Mean	£725	£570	£291	£208	£191	£178	£164	£147	£143
	SD	£3839	£3438	£2347	£2079	£1990	£1765	£1642	£1608	£1952
39	n	231 376	231 376	231 150	231 072	231 028	230 993	230 970	230 950	230 932
	Mean	£502	£453	£239	£175	£160	£160	£148	£127	£121
	SD	£3058	£2962	£2157	£1800	£1569	£1703	£1743	£1484	£1612
40	n	288 065	288 065	287 821	287 748	287 691	287 663	287 644	287 627	287 607
	Mean	£455	£394	£227	£164	£149	£142	£133	£122	£109
	SD	£2860	£2690	£2071	£1899	£1496	£1412	£1401	£1467	£1352
41	n	208 757	208 757	208 576	208 528	208 501	208 480	208 460	208 447	208 436
	Mean	£519	£358	£219	£158	£151	£154	£135	£120	£115
	SD	£2585	£2419	£1898	£1614	£1494	£1926	£1582	£1426	£1519
42	n	41 958	41 958	41 917	41 902	41 896	41 890	41 888	41 881	41 880
	Mean	£641	£391	£224	£159	£168	£152	£131	£118	£116
	SD	£2849	£2935	£1702	£1360	£2056	£1582	£1302	£1523	£1637

The TIGAR cohort excludes babies who died within their birth admission. As a result, for subsequent admissions all babies were alive at the beginning of the first year.

Full details on estimated lengths of stay for admissions subsequent to the birth admission, by year of follow-up, can be found in the online supplemental materials 4. Associated average hospital costs for these subsequent admissions are presented in table 4. Similar to birth admission costs, subsequent hospital admission costs decreased with increasing gestational age at birth, with the largest differences between gestational age groups observed during the first year after birth. Differences in hospital costs between gestational age groups diminished with increasing age, particularly during the first 2 years after birth, while children born extremely preterm (<28 weeks) and very preterm (28–31 weeks) still had higher hospital admission costs (£699 (95% CI £419 to £919) for <28 weeks; £434 (95% CI £305 to £563) for 28–31 weeks) during the eighth year of life compared with children born at 40 weeks (£109, 95% CI £104 to £114).

The mean cumulative hospital admission cost over 8 years after birth among children born extremely preterm was estimated at £80 559 (95% CI £79 238 to £82 019) per child, with most of the cost incurred during the first year after birth (£71 997 (95% CI £70 866 to £73 097)) (table 5). Children born at 40 weeks’ gestational age incurred the lowest 1-year, 5-year and 8-year cumulative hospital admission costs compared with other gestational age groups, with all cost differences being statistically significant (table 5). Even children born at 39 weeks had a higher 8-year cumulative hospital admission cost (£2085 (95% CI £2061 to £2107)) compared with those born at 40 weeks (£1894 (95% CI £1874 to £1912)). In the sensitivity analysis, after allocating all the non-critical care ward days to neonatal critical care for children born at ≤33 weeks, estimated total hospital admission costs were higher at these gestational ages, especially for the extremely preterm group where the 8-year total hospital admission cost increased to £119 044 (95% CI £117 350 to £120 909) per child.

**Table 5:** Total cumulative 1-year, 5-year and 8-year hospital cost (£) by gestational age at birth, estimated with the Kaplan-Meier sample-average estimator

	1-year total			5-year total			8-year total		
	Mean	95% CI	P value	Mean	95% CI	P value	Mean	95% CI	P value
<28	71 997	70 866 to 73 097	<0.0001	78 432	77 164 to 79 818	<0.0001	80 559	79 238 to 82 019	<0.0001
28–29	41 484	40 843, 42 135	<0.0001	44 846	44 097 to 45 584	<0.0001	46 499	45 705, 47 291	<0.0001
30–31	26 663	26 310 to 26 989	<0.0001	28 991	28 551 to 29 440	<0.0001	30 126	29 612 to 30 605	<0.0001
32	18 609	18 313 to 18 899	<0.0001	21 013	20 613 to 21 447	<0.0001	21 961	21 550 to 22 409	<0.0001
33	13 591	13 353 to 13 821	<0.0001	15 280	14 979 to 15 570	<0.0001	15 955	15 637 to16 257	<0.0001
34	9124	8918 to 9310	<0.0001	10 755	10 495 to 11 007	<0.0001	11 498	11 214, 11 769	<0.0001
35	5133	5001 to 5257	<0.0001	6474	6303 to 6638	<0.0001	7156	6967 to 7334	<0.0001
36	3351	3262 to 3438	<0.0001	4641	4523 to 4758	<0.0001	5266	5131 to 5399	<0.0001
37	1909	1866 to 1951	<0.0001	2943	2883 to 3002	<0.0001	3464	3402 to 3534	<0.0001
38	1294	1273 to 1315	<0.0001	2161	2128 to 2191	<0.0001	2612	2576 to 2646	<0.0001
39	955	940 to 970	<0.0001	1689	1669 to 1710	<0.0001	2085	2061 to 2107	<0.0001
40	848	837 to 861	Reference	1530	1512 to 1547	Reference	1894	1874 to 1912	Reference
41	877	865 to 889	<0.0001	1558	1540 to 1577	0.021	1928	1905 to 1950	0.013
42	1032	997 to 1066	<0.0001	1732	1685 to 1777	<0.0001	2096	2045 to 2148	<0.0001
Sensitivity analysis*									
<28	110 482	108 946 to 112 149	<0.0001	116 917	115 267 to 118 675	<0.0001	119 044	117 350 to 120 909	<0.0001
28–29	55 365	54 629 to 56 171	<0.0001	58 727	57 893 to 59 638	<0.0001	60 380	59 443 to 61 404	<0.0001
30–31	31 332	30 939 to 31 712	<0.0001	33 660	33 191 to 34 136	<0.0001	34 794	34 273 to 35 336	<0.0001
32	20 982	20 656 to 21 303	<0.0001	23 386	22 918 to 23 834	<0.0001	24 334	23 838 to 24 804	<0.0001
33	15 079	14 845 to 15 320	<0.0001	16 768	16 451 to 17 059	<0.0001	17 444	17 125 to 17 747	<0.0001

*Allocate all non-paediatric critical care birth admission days to neonatal critical care stay for gestational age <=33 weeks.

## Discussion

In this study, we have investigated hospital admission costs from birth to mid-childhood in England across the full range of gestational age at birth. We found that gestational age at birth is associated not only with birth admission hospital costs but also subsequent hospital admission costs up to age 8. The most common cause of subsequent admissions was infection.^8^ Children born extremely preterm were estimated to have high hospital admission costs throughout the first 8 years of life, with the majority of the costs incurred during the first year after birth.

Our cost estimates are in line with other studies of the costs of preterm birth in England. Khan *et al* reported similar birth admission hospital costs for 32–33 week moderately preterm born (£13 168) and 34–36 week late preterm born (£5463) children (2017–18 prices), based on a cohort in the East Midlands region of England.^14^ Mangham *et al* estimated the costs to the public sector over the first 18 years after birth using a decision-analytic model and reported neonatal care costs of £109 860 (2017–2018 price) for extremely preterm children.^11^ This is higher than our base case estimates (£53 144) when 2017 NNRD data are applied, but similar to the costs generated by our sensitivity analysis (£110 482) that assumed that the entire birth admission of children born at ≤33 weeks in TIGAR was spent in critical care.

This is, to our knowledge, the first study that uses a large national cohort to examine the association between gestational age at birth and hospital admission costs across the full spectrum of gestational age. The results of this study provide comprehensive estimates of hospital admission costs from birth to mid-childhood by gestational age at birth, which can act as a useful resource for clinical and budgetary service planning, and as data inputs for economic evaluations of preventive and treatment strategies for preterm birth.

The main strength of this study is the large sample size available for analysis, which provided a sufficient sample to estimate costs across the full spectrum of gestational age. The national coverage of the TIGAR cohort contrasts with the regional-based or clinic-based populations that provided a focus for previous studies.^10 14^ The linkage to routinely collected HES data accompanied by standardised costing approaches through HRG Groupers ensured an accurate estimation of hospital costs, as it based the estimation on more granular patient-level hospital activities and nationally recommended cost algorithms.

The main limitation of the study is that we were not able to estimate more granular neonatal and paediatric critical care costs for the TIGAR cohort as such estimation is not supported by the HES APC data; notably, critical care information was collected with insufficient quality in HES APC before 2008. A separate HES dataset covers adult critical care from 2008/2009, whereas data relating to neonatal or paediatric intensive care are collected through systems external to NHS Digital, which collects HES data.^17^ To address this issue, we conducted an evidence synthesis exercise and simulated cost estimates for neonatal or paediatric intensive care using aggregated data from the NNRD and PICANet. This allowed us to account for critical care costs in the analysis. In the case of PICANet, aggregate data were provided only by gestational week bands instead of a specific week. Therefore, we had to assume that the information provided for a specific band was the same across all weeks. This may have introduced some inaccuracies in our estimation of length of stay and associated costs for PCC. In addition, we were not able to obtain neonatal and paediatric critical care data over the same time coverage of the TIGAR cohort as such information does not date back to 2005–2006. This may have contributed to some of the differences between the total birth admission days observed in TIGAR and the neonatal and paediatric critical care days simulated using NNRD and PICANet data. Nevertheless, our sensitivity analysis that estimated costs using alternative assumptions about the ward stay during the birth admission of infants born either very or extremely preterm provides an upper bound for cost estimates for the birth admission.

It is also worth highlighting that the TIGAR cohort excluded children who died before discharge from their birth admission, which means that we have excluded a small group of extremely ill babies from our study. This suggests that our cost estimates should be viewed as conservative, particularly for the purposes of planning of neonatal services.

In conclusion, this study provides estimates of the association between gestational age at birth and hospital admission costs from birth to mid-childhood and disaggregates those estimates by gestational category and chronological year. The study results should act as a useful resource for future economic evaluations that focus on preventive and treatment strategies for preterm birth and inform resource allocation decisions.

## Data Availability

Data may be obtained from a third party and are not publicly available. The authors do not have permission to supply data or identifiable information to third parties, including other researchers, but the team at City, University of London has permission under regulation 5 of the Health Service (control of patient information) Regulations 2002 to analyse patient identifiable data for England and Wales without consent and create a research database that could be accessed by other researchers using the SRS at the ONS. The TIGAR team has permission under regulation 5 of the Health Service (control of patient information) Regulations 2002 to analyse these data. Anyone wishing to access the linked datasets for research purposes should apply via the CAG to the Health Research Authority to access patient identifiable data without consent and then to the ONS and NHS Digital. In the first instance, enquiries about access to the data should be addressed to Alison Macfarlane.
