# Supplementary material for: Gestational age and hospital admission costs from birth to childhood: a population-based record linkage study in England
Source: Arch Dis Child Fetal Neonatal Ed. 2023 Feb 9;108(5):485–91. doi: 10.1136/archdischild-2022-324763 (PMC10447377; doi:10.1136/archdischild-2022-324763)
Supplement: Supplementary data [file fetalneonatal-2022-324763supp001.pdf]

Supplementary material

Title: Gestational age and hospital admission costs from birth to childhood: a population based record linkage study in England

Authors: Xinyang Hua, Stavros Petrou, Victoria Coathup, Claire Carson, Jennifer J Kurinzcuk, Maria A Quigley, Elaine Boyle, Samantha Johnson, Alison Macfarlane, Oliver Rivero-Arias

**Content**	**Page**
Supplementary material 1. Methods to estimate neonatal and paediatric critical care cost in birth admission	1-4
Supplemental material 2. Summary on Grouping results	5-7
Supplemental material 3. Summary on Reference Cost matching	8
Supplementary materials 4. Statistics for subsequent admissions	9-10

Supplementary material 1. Methods to estimate neonatal and paediatric critical care cost in birth admission

1.1 Variables related to neonatal/paediatric critical care stay within the Hospital Episode Statistics Admitted Patient Care (HES APC) https://digital.nhs.uk/data-and-information/data-tools-and-services/data-services/hospital-episode-statistics

The following variables within the HES APC provide information on neonatal critical care stay which potentially can be used in the TIGAR cohort (babies born between January 2005 and December 2006):

- Variable "neocare" which indicates level of neonatal care in each hospital episode. In specific --

  0 = Normal care
  1 = Special care
  2 = High dependency intensive care
  3 = Maximal intensive care
  8 = Not applicable
  9 = Not known

- Before 1st April 2008, there was a section about "augmented/critical care" within the HES APC, including a set of variables that can be used to estimate the number of days spent in different critical care units (Neonatal/Paediatric/Adult) in each episode. Those variables are often missing. When all the variables in the section were missing, we viewed them as with no critical care stay in the episode.

To investigate the reliability of these variables to estimate the costs of neonatal/paediatric critical care stay for the birth admission, we tried to use them to identify critical care study in the extremely preterm baby group (gestational age <= 27 week) in our cohort. The results showed that among the birth episodes for those extremely preterm babies:

Distribution of the "neocare" variable:

	Percent
0=Normal care	17.7
1=Special care	14.2
2=Level 2 IC (high dep)	5.5
3=Level 1 IC (max)	33.3
8=Not applicable	15.3
9=Not known	14.0
Total	100.0

Percentage of episodes with critical care, estimated based on the "augmented/critical care" section

	Percent
Without critical care (missing values)	94.6
With critical care	5.4
Total	100.0

53% of the birth episodes using the “neocare” variable (neocare = 1,2 and 3) and 5.4% using the “augmented/critical care” section were identified as with critical care. Both of these two figures were considered low in this group of babies who were born less than <= 27 weeks.

As a result, we’ve decided that these variables could not be used for the analysis to estimate neonatal critical care costs.

Hua X, *et al. Arch Dis Child Fetal Neonatal Ed* 2023; 108:1–7. doi: 10.1136/archdischild-2022-324763

1.2 Methods to simulate neonatal critical care (NCC) and paediatric critical care (PCC) stay and costs in birth admission

To account for costs spent in critical care units, we requested aggregated data on the number of babies admitted to neonatal critical care (from NNRD) and paediatric critical care (from PICANet), and the average days a baby spent in different levels of critical care during the birth admission by sex and gestational age at birth, in England. The average number of days was estimated for babies who were alive when discharged from either neonatal or paediatric units to maintain consistency with the TIGAR sample. We requested NNRD and PICANet data for 2017 as granular information on the level of care required to generate Healthcare Resource Group (HRG) codes, which was essential for the costing, was not available in the PICANet data in earlier years.

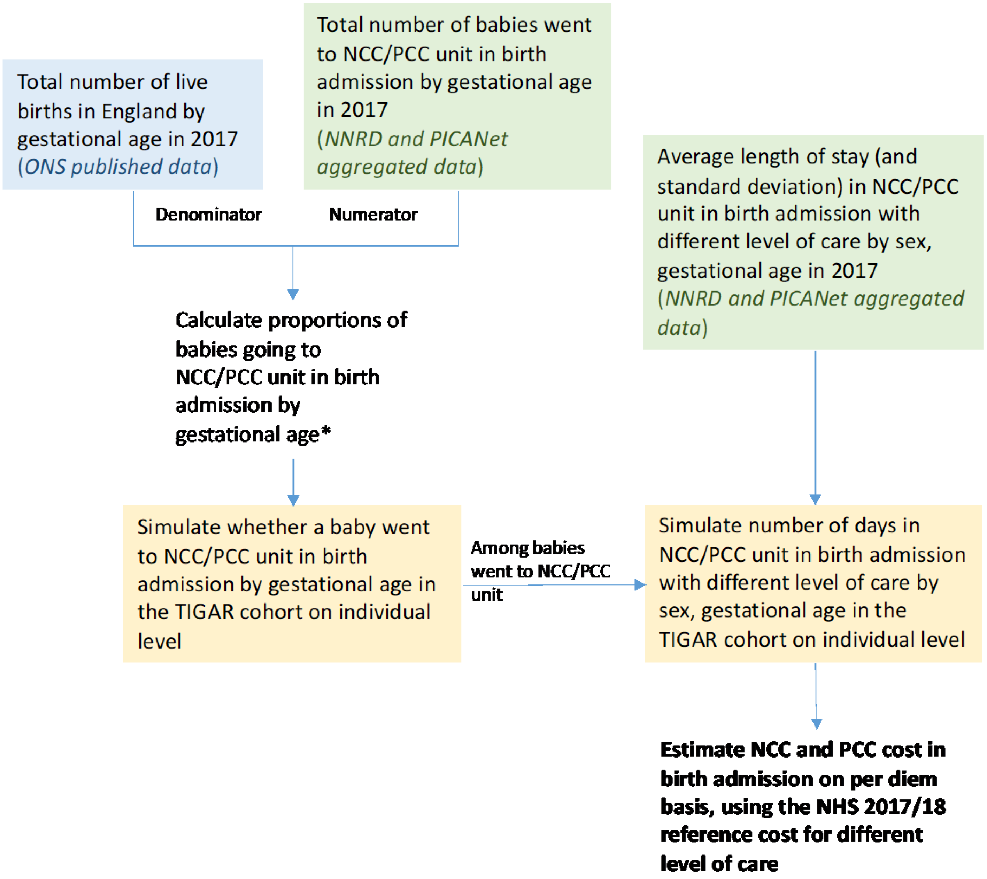

***for GA <=33 week, assume 100% went to NCC. This is higher than the %s calculated using the ONS and NNRD aggregated data and can be explained by high risk of live births dying in the delivery room before being able to be transferred to NCC in these very preterm groups (Costeloe et al. 2012. BMJ). In TIGAR all babies were alive when discharged from birth admission, so we assumed 100% of these early term babies in TIGAR went to NCC unit.**

1.3 Methods to adjust non-critical care cost

First calculate average total NCC and PCC days during the birth admission by gestational age among the entire TIGAR cohort.

Then for each gestational week group, adjust the non-critical care (ward) cost during birth admission as:

$$Days_{GAx.wards} = Days_{GAx.hosp} – Days_{GAx.NCC} - Days_{GAx.PCC}$$

$$Cost_{GAx.wards} = Cost_{GAx.hops} * Days_{GAx.wards} / Days_{GAx.hosp}$$

where—

$Days_{GAx.hosp}$ is the average total hospital days during birth admission for gestational age group X;

$Days_{GAx.NCC}$ is the average total NCC days during birth admission for gestational age group X;

$Days_{GAx.PCC}$ is the average total PCC days during birth admission for gestational age group X;

$Cost_{GAx.hops}$ is the average total hospital cost for gestational age group X when assuming all the hospital stay were in wards, calculated from the Grouper (https://digital.nhs.uk/services/national-casemix-office/downloads-groupers-and-tools/costing---hrg4-2018-19-reference-costs-grouper)

Supplemental material 2. Summary on Grouping results

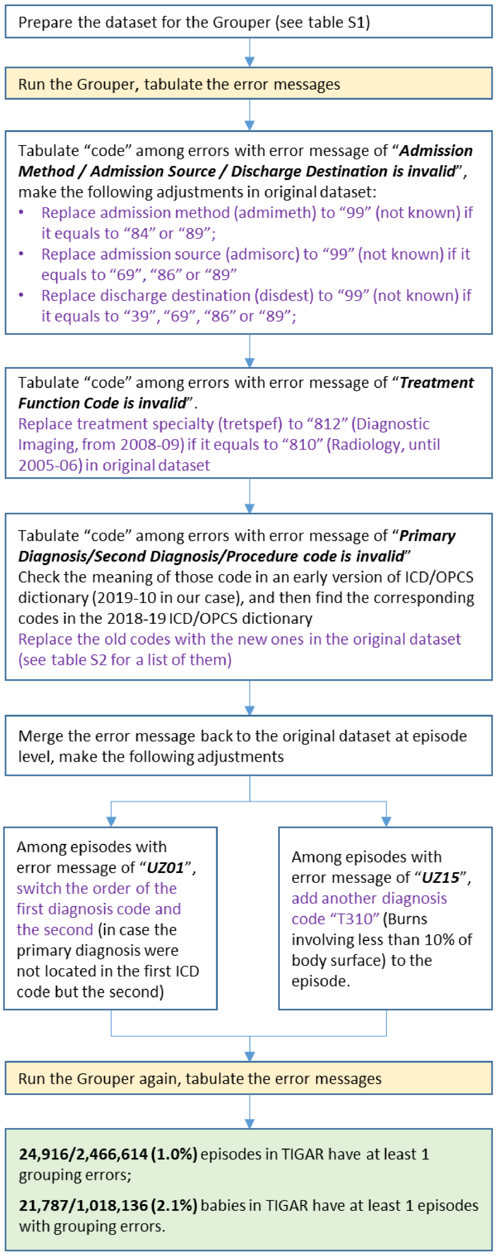

Error message	Count
Admission Method / Admission Source / Discharge Destination is invalid	98
Treatment Function Code is invalid	66
Primary Diagnosis / Second Diagnosis / Procedure code is invalid	137,164
UZ01 - Invalid Primary Diagnosis	10,460
UZ02 - Poorly Coded Primary Diagnosis	8,745
UZ05 - Invalid procedure for Casemix grouping purposes	252
UZ06 - Poorly coded procedure for Casemix grouping purposes	954
UZ15 - Burns primary diagnosis code of unspecified body region or with no subsequent total body surface area(TBSA) code	2,114
**Total**	**159,853**

Error message	Count
UZ01 - Invalid Primary Diagnosis	2,268
UZ02 - Poorly Coded Primary Diagnosis	21,613[*]
UZ05 - Invalid procedure for Casemix grouping purposes	245[§]
UZ06 - Poorly coded procedure for Casemix grouping purposes	954
UZ15 - Burns primary diagnosis code of unspecified body region or with no subsequent total body surface area(TBSA) code	55
**Total**	**25,135[#]**

*Number increased since more poorly coded (too vague) diagnosis were able to be recognized by Grouper after updated the old ICD codes to the new ones. For example "R69X" become able to be recognized by the Grouper after adapting from "R69", but it's too vague for the Grouper to allocate a HRG code for it (Unknown and unspecified causes of morbidity)

§Number slightly decreased since after updated the old procedure codes to new ones, some episodes got a related major procedure from one of the procedure codes

Some episodes had more than one errors, so this number is larger than the total number of episodes with grouping errors

Variables required for the 2018/19 Grouper

Variable	Meaning
PROCODET	Provider code of treatment
PROVSPNO	Hospital provider spell number
EPIORDER	Episode order within the current spell
STARTAGE	Age at start of episode (whole year rounded down)
SEX	Sex of patient
CLASSPAT	Patient classification (e.g. day cases, ordinary admissions, etc.)
ADMISORC	Source of admission
ADMIMETH	Method of admission
DISDEST	Destination on discharge
DISMETH	Method of discharge
EPIDUR	Episode duration (days)
MAINSPEF	Main specialty
TRETSPEF	Treatment specialty
NEOCARE	Neonatal level of care
DIAG_01	Primary diagnosis (ICD)
DIAG_02 – NN	Secondary diagnosis (ICD)
OPER_01 – NN	Procedure (OPCS)

*Critical care days is an optional field in the Grouper which can help to adjust the non-critical care episode duration. No episode level critical care days were available in our TIGAR data. We have left this field blank (presume 0 critical care days) when running the Grouper. Adjustment on birth admission non-critical care costs were made on baby level based on the simulated neonatal critical care days in birth admission (see Supplementary material 3 for details).

Old diagnosis and procedure codes in TIGAR and the corresponding new codes

**Invalid (old) codes for 2018/19 Grouper**	**New codes for 2018/19 Grouper**	**Invalid (old) codes for 2018/19 Grouper**	**New codes for 2018/19 Grouper**
ICD codes			
R69	R69X	N62	N62X
A09X	A090	R71	R71X
N180	N185	A86	A86X
D760	C966	D67	D67X
K350	K352	I840	K649
Q314	Q315	I847	K649
K359	K358	L52	L52X
R500	R509	L80	L80X
P38	P38X	N188	N182
P90	P90X	R36	R36X
R21	R21X	R72	R72X
R501	R509	R95	R959
I846	K644	Z225	Z228
K351	K353	Z33	Z33X
N47	N47X	B07	B07X
K85X	K859	B49	B49X
H547	H549	B99	B99X
C80X	C809	C850	C859
C961	C968	D62	D62X
I48X	I489	D70	D70X
I849	K649	E58	E58X
Q02	Q02X	E86	E86X
P77	P77X	G01	G01X
I845	K649	G35	G35X

R14	R14X	G903	G904
L89X	L899	I10	I10X
R17	R17X	I81	I81X
D66	D66X	I99	I99X
L22	L22X	J81	J81X
M725	M726	J90	J90X
R11	R11X	L84	L84X
R33	R33X	L89	L899
N44	N44X	L97	L97X
I848	K649	N12	N12X
P75	P75X	N63	N63X
P95	P95X	N86	N86X
Q356	Q359	P93	P93X
I38	I38X	R02	R02X
I842	K649	R13	R13X
I843	K649	R34	R34X
R31	R31X	R35	R35X
D752	D759	R51	R51X
H55	H55X	R53	R53X
A90X	A979	R55	R55X
C836	C839	R58	R58X
J22	J22X	R80	R80X
P53	P53X	R81	R81X
R91	R91X	R98	R98X
R95X	R959	T68	T68X
I48	I489	Y69	Y69X
L00	L00X	Y95	Y95X
N19	N19X	Z21	Z21X
P60	P60X	U800	U820
R05	R05X	U801	U821
R18	R18X	U808	U828
A09	A090	U810	U830
B24	B24X	U818	U831
D45	D45X	U88X	U837
I841	K649	U898	U838
I844	K649	U899	U839
M723	M726		
OPCS codes			
X632	X674		
X633	X677		
X634	X674		
X642	X682		
X643	X683		
X648	X688		
X649	X689		
S522	S521		
S524	S523		

Supplemental material 3. Summary on Reference Cost matching

1. Due to the delay in publishing the 2018/19 reference cost by the NHS Improvement, the reference costs we used in this study were drawn from schedules from the previous year (2017/18 reference costs). As a result, some HRGs generated by the 2018/19 Grouper Software cannot be found in the 2017/18 reference cost schedules. We have firstly detected these new HRGs in TIGAR and replaced them with the corresponding older ones:

New HRGs in the 2018/19 Grouper	Corresponding old HRGs in 2017/18
AB25Z	AB17Z
AB26Z	AB17Z
AB27Z	AB19Z
AB28Z	AB19Z
BZ74Z	BZ74B
FF42Z	FF42B
LB81Z	LB29C / LB29D (based on age)
RD60Z	RD28Z

2. Matching the core HRGs to core HRG reference costs (2,466,614 episodes in total)

Matched by HRG, admission type and treatment specialty	1,403,649
Matched by HRG, admission type	254,450
Matched by HRG only	1,288
Attach a zero cost (PB03Z, LA97B, PB13A/B/C/D*)	782,311
Attach a length of stay specific average cost for episodes with a grouping error (UZ01Z)	24,916

*For PB13A/B/C/D, attach zero cost at episode level and manually attach cystic fibrosis year of care currencies at baby level

3. Matching the core HRGs to excess bed day reference costs (2,466,614 episodes in total)

Episodes with no excess bed days (zero cost)	2,349,169
Matched by HRG, admission type and treatment specialty	71,764
Matched by HRG, admission type	35,381
Matched by HRG only	171
Attach a zero cost (PB03Z, WF01A/WF02A, PB13A/B/C/D)	10,120
For some HRGs, there's no activities in the current year to the derive reference cost -- use reference cost in previous years (then inflate) / use reference cost for a close HRG	9

4. Matching the unbundled HRGs to reference cost (113,027 unbundled HRGs generated in total)

Matched by HRG	98,354
Attach a zero cost for episodes with a grouping error (UZ01Z)	747
High cost drugs with no reference cost at HRG level – use the weighted average cost across all high cost drugs	13,926

Supplementary materials 4. Statistics for subsequent admissions

Length of stay in subsequent admissions (days) by year of follow-up and gestational age

		Year 1	Year 2	Year 3	Year 4	Year 5	Year 6	Year 7	Year 8
<28	n	1,730	1,710	1,705	1,701	1,697	1,695	1,695	1,691
	Mean	6.8	3.2	1.5	1.0	0.6	0.5	0.4	0.5
	SD	18.2	17.7	9.1	5.8	4.7	4.7	3.8	7.2
28-29	n	2,089	2,071	2,069	2,067	2,067	2,066	2,065	2,064
	Mean	3.7	1.5	0.6	0.5	0.3	0.4	0.4	0.3
	SD	12.1	10.2	3.7	4.4	3.0	3.7	4.8	3.7
30-31	n	3,227	3,218	3,217	3,214	3,213	3,212	3,211	3,211
	Mean	2.6	0.7	0.5	0.3	0.3	0.3	0.2	0.3
	SD	8.6	3.9	6.0	2.2	2.8	3.8	2.2	4.1
32	n	2,656	2,646	2,645	2,644	2,642	2,642	2,641	2,639
	Mean	2.3	0.8	0.5	0.4	0.3	0.2	0.2	0.1
	SD	8.9	5.6	5.7	3.4	3.4	2.5	2.2	1.5
33	n	4,050	4,026	4,021	4,018	4,017	4,016	4,015	4,015
	Mean	1.6	0.7	0.3	0.2	0.2	0.2	0.1	0.1
	SD	8.1	5.4	3.2	2.2	1.8	1.3	0.9	0.8
34	n	7,292	7,255	7,247	7,244	7,242	7,240	7,240	7,240
	Mean	1.7	0.7	0.4	0.3	0.1	0.2	0.2	0.1
	SD	9.6	6.9	4.7	3.0	1.2	3.0	2.6	1.8
35	n	11,663	11,614	11,601	11,593	11,590	11,588	11,588	11,583
	Mean	1.3	0.5	0.3	0.2	0.2	0.2	0.1	0.2
	SD	8.6	5.2	3.2	2.5	3.2	2.7	2.7	3.9
36	n	23,346	23,273	23,255	23,249	23,246	23,239	23,237	23,235
	Mean	1.2	0.4	0.3	0.2	0.2	0.1	0.1	0.1
	SD	7.9	4.0	4.3	3.0	3.2	2.4	2.7	2.6
37	n	54,001	53,889	53,865	53,845	53,836	53,824	53,818	53,812
	Mean	0.9	0.3	0.2	0.2	0.1	0.1	0.1	0.1
	SD	5.6	3.4	2.5	2.9	1.8	2.0	1.4	2.2
38	n	137,926	137,711	137,654	137,616	137,596	137,575	137,563	137,553
	Mean	0.6	0.3	0.2	0.1	0.1	0.1	0.1	0.1
	SD	4.5	2.7	2.5	2.2	1.6	1.4	1.6	1.6
39	n	231,376	231,150	231,072	231,028	230,993	230,970	230,950	230,932
	Mean	0.5	0.2	0.1	0.1	0.1	0.1	0.1	0.1
	SD	3.9	2.4	1.9	1.5	1.6	1.7	1.4	1.4
40	n	288,065	287,821	287,748	287,691	287,663	287,644	287,627	287,607
	Mean	0.4	0.2	0.1	0.1	0.1	0.1	0.1	0.1
	SD	3.3	2.3	2.2	1.4	1.3	1.3	1.2	1.1
41	n	208,757	208,576	208,528	208,501	208,480	208,460	208,447	208,436
	Mean	0.4	0.2	0.1	0.1	0.1	0.1	0.1	0.1
	SD	3.0	2.2	1.8	1.5	1.4	1.2	1.3	1.3
42	n	41,958	41,917	41,902	41,896	41,890	41,888	41,881	41,880
	Mean	0.4	0.2	0.1	0.1	0.1	0.1	0.1	0.1
	SD	4.1	2.1	1.6	2.8	1.4	1.2	1.4	1.9

The TIGAR cohort excludes babies who died within their birth admission. As a result, for subsequent admissions all babies were alive at the beginning of the first year.
